# Application and effects of an early childhood education machine on analgesia and sedation in children after cardiothoracic surgery

**DOI:** 10.1186/s13019-021-01490-2

**Published:** 2021-05-01

**Authors:** Li-Li Chen, Yu-Qing Lei, Jian-Feng Liu, Hua Cao, Xian-Rong Yu, Qiang Chen

**Affiliations:** 1 Department of Cardiac Surgery, Fujian Maternity and Child Health Hospital, Affiliated Hospital of Fujian Medical University, Fuzhou, China; 2 Fujian Key Laboratory of Women and Children’s Critical Diseases Research, Fujian Maternity and Child Health Hospital, Fuzhou, China; 3 Fujian Branch of Shanghai Children’s Medical Center, Fuzhou, China; 4 Fujian Children’s Hospital, Fuzhou, China

**Keywords:** Children’s early education machine, Congenital heart disease, Sedation, Analgesia, Negative emotion

## Abstract

**Objective:**

To study the effect of an early childhood education machine on sedation and analgesia in children after cardiothoracic surgery.

**Methods:**

A prospective randomized controlled study was conducted in a provincial hospital in China. Fifty-two patients (aged from 1 to 5 years) underwent cardiothoracic surgery (including: ventricular septal defect, patent ductus arteriosus, atrial septal defect, pulmonary stenosis, pulmonary sequestration and congenital cystic adenomatoid lung malformation) were divided into the study group (*n* = 26) and the control group (*n* = 26). The patients in the study group underwent intervention with an early childhood education machine (uniform type) in addition to routine standard treatment and nursing, while the patients in the control group only received routine standard treatment and nursing. Richmond agitation sedation score (RASS) and face, legs, activity, cry, consolability (FLACC) score of all of the patients were evaluated, and the negative emotions (self-rating anxiety scale (SAS) score and self-rating depression scale (SDS) score) of the parents of the two groups were compared.

**Results:**

There was no significant difference in the general clinical data between the two groups. The RASS and FLACC scores in the study group were significantly lower than those in the control group, and the SAS and SDS scores of the parents in the study group were significantly lower than those in the control group.

**Conclusion:**

The application of an early childhood education machine for children after cardiothoracic surgery can effectively reduce postoperative agitation, improve sedation and analgesia of the patients, and ease the pessimistic mood of the patients’ parents.

## Introduction

Congenital heart disease and congenital diseases of the esophagus and lungs are the most common congenital diseases in children. Surgical repair is a traditional method for treating these conditions [1]. Although the surgical technique is mature, the operation requires a median or thoracic incision, and most cardiac surgery requires a hypothermic cardiopulmonary bypass. Thus, the incidence of postoperative restlessness in children is high. Recently, minimally invasive technology has been widely used in cardiothoracic surgery, which has the advantages of a small incision, less trauma, no need for cardiopulmonary bypass, short operation time, and a quick recovery [2, 3]. However, some patients still need traditional surgery, and postoperative restlessness is inevitable. Due to the postoperative unfamiliar surroundings and the painful incision, children often feel fear and anxiety, producing negative emotions in their families, and reducing the parents’ satisfaction with treatment [4–6]. Therefore, it is essential to reduce the pain, restlessness, and anxiety of children after cardiothoracic surgery. Early childhood education machines are prevalent in China. With their unique working model, they can partially replace busy parents and become critical growth partners for children. They can be placed on the patient’s bed, and distract the children’s attention and play a sedative and analgesic role through music and videos from the machine. This study summarized our experience of using early childhood education machines to relieve the restlessness, anxiety, and pain of children after cardiothoracic surgery in our hospital.

## Methods and material

According to the preliminary research results and statistical calculations, the total research object was set as 52. Between January 2020 and May 2020, the clinical data of 52 children who underwent cardiothoracic surgery in our hospital were collected. The eligible patients were randomly divided into the study group (early childhood education machine intervention group) and the control group (routine treatment group) by random number generation with a computer. Inclusion criteria were as follow: (1) the age ranged from 1 to 5 years; (2) all children diagnosed with cardiothoracic diseases by echocardiography or chest CT examination before the surgery, without any other organ deformities, no history of a previous operation, and no other systemic diseases; (3) the tracheal tube was removed successfully after the operation; (4) the routine drug treatment plans of the two groups were the same; (5) the parents knew about the aim of the research and participated voluntarily, all patients’ parents signed an informed consent form before the study. Exclusion criteria were as follow: (1) patients needed staged surgical correction, or palliative surgery, (2) patients needed mechanical ventilation for more than 24 h after surgery; (3) postoperative cardiac insufficiency alone or combined with other organ dysfunction; (4) the patient was complicated with vision, hearing, or neurological dysfunction; (5) the parents of the patients refused to participate in this study.

The patients in the control group were treated with routine pediatric analgesic drugs after surgery. They were then given routine nursing care, including posture, treatment nursing, condition observation, medication effect observation, diet and psychological nursing. A standard protocol of 2.0 μg/kg sufentanil, 0.2 mg/kg tropisetron, and 100 ml physiological saline was used with the speed of 2 ml/h for the first postoperative 48 h. In addition to the interventions described above, the patients in the study group were provided with the early childhood education machine when they were awake. The early childhood education machine was usually start at a certain time (6, 9, 12, 15 and 18 o’clock) or when the patient was agitation for about 30 min at a time, and the total use time was controlled in no more than 3 h each day. If the patients were in a state of sleep, we would cancel the use of the early education machine. We usually placed the machine on either side of the patient’s head. If the patient could take a semi-decubitus position, we would put the machine on a small table on the hospital bed. The independent researchers stayed with the patient throughout the intervention process and chose appropriate music and videos according to their speech feedback and facial expressions.

The same type of early education machine manufactured by a Chinese company was used in this study, whose applicable age was 1–6 years old. The early childhood education machine’s selected material strictly complied with international standards and had obtained a national health certification. The machine had an integrated shell, which was covered with anti-static fabric, was nontoxic, lead-free, did not fade, and had no sharp edges or corners. The machine was an electronic educational product specially designed for children’s early education to promote children’s interest in learning. According to the children’s age and the communication of their parents, we chose the music or video that was appropriate for the patient, and store them in the machine. English and Chinese literacy, mathematical logic, potential development, natural common sense, parent-child interactions, entertainment, morality, and eight other areas were integrated into each topic. The researchers maintained close communication with the patients’ families, informed them about the patients’ disease status, explained the purpose of using the early childhood education machine to the parents, and obtained their cooperation and understanding.

The patients’ general data, including age, gender, weight, operation time, the distribution of disease spectrum, the incision location and the parents’ general data were collected in the Table 1. The children were all evaluated with the Richmond agitation sedation score (RASS) and the face, legs, activity, cry, consolability (FLACC) scale. Besides, the negative emotions (self-rating anxiety scale (SAS) and self-rating depression scale (SDS)) of the two groups’ parents were also assessed and recorded. The above scale was used on the second day after the operation.

**Table 1:** Comparison of the general data between the two groups

	Early childhood education machine group	Control group	***P*** value
**Patients**			
**Boys/girls**	13/13	15/11	0.578
**Age (year)**	3.31 ± 1.03	3.40 ± 1.01	0.613
**Height (cm)**	92.81 ± 11.80	92.56 ± 13.78	0.446
**Weight (Kg)**	15.49 ± 6.87	15.35 ± 4.50	0.319
**Disease**			
**Ventricular septal defect**	12	10	0.965
**Patent ductus arteriosus**	1	2	
**Atrial septal defect**	4	3	
**Pulmonary stenosis**	2	3	
**Pulmonary sequestration**	4	5	
**Congenital cystic adenomatoid lung malformation**	3	3	
**Operation time (h)**	2.96 ± 0.61	3.16 ± 0.46	0.874
**Operation with cardiopulmonary bypass**	18	16	0.560
**Operation without cardiopulmonary bypass**	8	10	
**Type of incision**			
**Median incision**	18	16	0.772
**Left thoracic incision**	4	6	
**Right thoracic incision**	4	4	
**The length of ICU stay (h)**	9.8 ± 4.61	10.8 ± 5.32	0.412
**The length of hospital stay (d)**	5.5 ± 1.12	5.8 ± 1.35	0.731
**Parents**			
**Age (year)**	29.64 ± 4.23	28.75 ± 5.36	0.658
**Education level**			
**Under high school**	5	5	0.936
**High school**	9	7	
**Junior college**	8	9	
**Bachelor degree or higher**	4	5	
**Living condition**			
**Rural area**	16	14	0.575
**City**	10	12	

The RASS was a reliable and effective scale to evaluate critically ill children’s sedation effect [7, 8]. The RASS scale was divided into ten grades with a score ranging from + 4 to − 5, representing the patient’s degree of sedation from “aggressiveness” to “coma”. Each score corresponded to a state of consciousness. Merkel especially developed the FLACC scale for children’s postoperative analgesia score in 1997 [9, 10]. The researchers needed to observe the children for 1 to 15 min, and their pain scores were assessed by a quantitative table and the observed children’s condition. Each item was scored 0–2, and the total score was the sum of five terms.

Parents’ negative emotions were evaluated with a SAS and a SDS developed by Zung in 1971 and 1965 [11, 12]. There were 20 items in each of the two scales, and each symptom was divided into four grades. The ascending scores were 1, 2, 3, and 4 based on the occurrence frequency of the positive symptoms, and the reverse scores were 4, 3, 2, and 1 based on the occurrence frequency of the negative symptoms. The SDS and SAS’s main statistical index was the total score, which was the sum of the scores of the 20 items, that was, the rough score, and the standard score was equal to the integer part of the rough score multiplied by 1.25. According to the Chinese norm, the SDS standard score’s cut-off value was 53, and the SAS standard score was 50.

Before the SAS and SDS self-assessment, the volunteers informed the parents about the method of filling in the whole scale and the meaning of each question, and then the parents made an independent self-assessment that was not influenced by anyone. The time frame of the assessment was the actual feeling of the self-reviewer over the past week. If the parents’ educational level were too low to understand the SAS and SDS contents, a volunteer would read it and allowed them to make the assessment alone. At the end of the evaluation, the volunteers carefully checked the evaluation results and reminded the self-reviewers to fill in missing information and not to repeat the evaluation on the same item.

Continuous data were presented as the mean ± standard deviation and range. All the continuous data were tested for normal distribution, and the results were confirmed to normal distributions. Clinical parameters between the two groups were compared with the independent samples t-test. The χ2 or Fisher’s test was used for the categorized variables. A *p*-value of < 0.05 was defined as a significant difference.

## Results

In terms of patients, there were no significant statistical differences between the two groups in gender, age, height, weight, operation time, the length of ICU and hospital stay. Similarly, there was no significant difference in the distribution of disease spectrum, operation method and the incision location between the two groups. There was also no statistically significant difference between the two groups in terms of parental education or residence (*P* > 0.05, Table 1).

The RASS and FLACC scores in the early childhood education machine intervention group were significantly better than those in the control group (RASS score: *P* = 0.032, FLACC score: *P* = 0.035), as shown in Table 2. The SAS score and SDS score of the study group were significantly lower than those of the control group, and there was a statistically significant difference (SAS score: *p* = 0.028, SDS score: *p* = 0.031). (Table 3) There was no significant difference in heart rate and mean arterial pressure between the two groups before the intervention. However, the heart rate and mean arterial pressure in the study group were significantly lower than those in the control group after the intervention (Table 4).

**Table 2:** Comparison of postoperative RASS score and FLACC score between the two groups

	Early childhood education machine group	Control group	***P*** value
**RASS score**	0.62 ± 0.70	1.85 ± 0.85	0.032
**FLACC score**	1.58 ± 0.54	3.12 ± 0.65	0.035

**Table 3:** Comparison of negative emotion of the patients’ parents between the two groups

	Early childhood education machine group	Control group	***P*** value
**SAS score**	50.46 ± 8.31	63.27 ± 8.23	0.028
**SDS score**	52.50 ± 7.06	62.54 ± 9.92	0.031

**Table 4:** Comparison of hemodynamic data between the two groups

	Early childhood education machine group	Control group	***P*** value
**Before intervention**			
**Heart rate (beat/min)**	105.56 ± 18.65	108.64 ± 16.12	0.643
**Mean arterial pressure (mmHg)**	86.63 ± 13.74	88.86 ± 14.18	0.716
**After intervention**			
**Heart rate (beat/min)**	103.13 ± 11.25	112.64 ± 12.22	0.039
**Mean arterial pressure (mmHg)**	90.24 ± 12.21	100.45 ± 13.28	0.031

## Discussion

Congenital heart disease and congenital diseases of the lungs, esophagus, and mediastinum are common conditions in children that need cardiothoracic surgery. In recent years, minimally invasive surgery, percutaneous device technology, and video-assisted thoracoscopy have been rapidly developed and widely used in cardiothoracic surgery, and most patients can receive these treatments [13, 14]. However, some patients still need traditional median and transthoracic incisions. Due to the surgical incision and trauma, perioperative pain is an inevitable result. Pain is a predictable postoperative experience, but inadequate analgesic therapy is a common reason that could have far-reaching adverse effects, including physical and psychological changes, an increased incidence of postoperative complications, and increased pain management costs [15, 16]. In recent years, with the development of medical care and increased expectations by children’s families, it is more important than ever to carry out treatment and nursing of postoperative restlessness and pain in children after cardiothoracic surgery.

Analgesia and sedation therapy is the essential treatments after pediatric cardiothoracic surgery. The sedative and analgesic drugs commonly used in pediatric cardiothoracic surgery include opioids, benzodiazepines^,^ and dexmetomidine [6, 17]. Besides, a variety of effective non-pharmacological analgesic treatment regimens have been adopted in the clinic. At present, there are many articles about the effect of music therapy on surgical analgesia [18, 19]. Considering nearly the same mechanism, we had the reason to believe that an early education machine also had a specific analgesic and sedative effect on children after cardiothoracic surgery.

In this study, an early childhood education machine was used as an intervention, which is generally suitable for children aged 1–6 years old. The literature has shown that artificial intelligence has begun to impact all aspects of the human condition, including medical practice and teaching [20]. Attracting children’s attention in the form of music or video makes most children like the early education machine’s mode. The research on early childhood education machines in foreign countries has a history of nearly 30 years. In western countries, early education machines are mainly used as a toy. Although there are many years of research experience in foreign countries, there are still no reports on early education machines in children after pediatric surgery.

RASS is a sedation effect evaluation tool recommended by many guidelines and has apparent advantages in evaluating critically ill children’s sedation effect. FLACC shows good reliability and validity and can be a useful tool for postoperative pain assessment in preschool children. SDS and SAS can directly reflect the subjective feelings of patients with depression and its changes in treatment, which has been widely used in outpatient and inpatient screening, emotional state assessment, investigation, scientific research and so on. The results of this study showed that the RASS and FLACC scores in the study group were significantly lower than those in the control group, and the SAS and SDS scores of the parents in the study group were also lower than those in the control group, which indicated that the intervention methods we adopted affected the analgesia and sedation of the children after cardiothoracic surgery, and improved the mood of the parents.

The lovely cartoon images, the design of its appearance and color, and the machine’s integrated voice recognition and visual analysis technology could simulate the experience of human voices and promote interactions with the machine. This could allow the children to experience psychological pleasure and relaxation, bring more joy, and play a positive role in preventing restlessness and relieving pain. Meanwhile, this could also be an easy way to attract the children’ attention and avoid anxiety and fear caused by their unfamiliar environment. At the same time, due to the relief of the patient’s symptoms and the improvement of the patient’s compliance, the negative emotions of the patient’s parents would be reduced, and the satisfaction of treatment would be increased accordingly.

There were still some limitations in this study, such as the small sample size and the restriction to children with cardiothoracic diseases. Therefore, it might be unreasonable for our results to be applied to other surgical patient groups. Besides, many subjective evaluation indicators had used this study, which might easily lead to selection deviation. Although the results of our study suggested that the early childhood education machine had certain effects on postoperative sedation and analgesia in children underwent cardiothoracic surgery, we could not provide the accurate onset time of the sedation and analgesia effect of the early childhood education machine on those patients. There might be different results in different units, regions, races, diseases, and even different age groups. In the future, it is necessary to consider a variety of factors and study larger samples with longer follow-up to validate the conclusion.

## Conclusion

As a non-pharmacological analgesic treatment, an early childhood education machine can effectively reduce the incidence of restlessness and pain in children and avoid negative emotions in parents after a toddler undergoes cardiothoracic surgery. Therefore, this method has achieved a good clinical effect and is worthy of clinical application.

## Data Availability

Data sharing not applicable to this article as no data sets were generated or analyzed during the current study.

## Abbreviations

RASS: Richmond agitation sedation score
FLACC: The face, legs, activity, cry, consolability scale
SAS: Self-rating anxiety scale
SDS: Self-rating depression scale
